# Does the thoracolumbar kyphosis secondary to ankylosing spondylitis affect the iliac trajectory of S2AI screw?

**DOI:** 10.1186/s12891-022-05140-z

**Published:** 2022-03-02

**Authors:** Xiao-lin Zhong, Bang-ping Qian, Ji-chen Huang, Bin Wang, Yong Qiu

**Affiliations:** 1 Division of Spine Surgery, Department of Orthopedic Surgery, Affiliated Drum Tower Hospital, Medical School of Nanjing University, Zhongshan Road 321, Nanjing, 210008 China; 2 Medical School of Nanjing University, Nanjing, China

**Keywords:** Second sacral alar iliac (S2AI) screw, Sacroiliac joint, Three-dimensional computed tomography (3DCT) imaging, Ankylosing spondylitis, Thoracolumbar kyphosis

## Abstract

**Background:**

The study aimed to evaluate the influence of thoracolumbar kyphosis secondary to ankylosing spondylitis (AS) on parameters of S2AI trajectory and to compare the ideal S2AI trajectory with those of the non-deformity patients with AS, sagittal deformity patients without AS, and the normal population reported in literatures.

**Methods:**

Sagittal parameters including global kyphosis (GK), pelvic tilt (PT) and sacral slope (SS) were measured. Besides, according to the simulated ideal S2AI trajectory on the CT images, trajectory parameters were measured including Sag angle, Tsv angle, Max-length, Sacral distance and Iliac width. Starting-point parameters were also measured including PSIS distance, Skin distance, Iliac wing and S2 midline.

**Results:**

Ninety-four AS-related thoracolumbar kyphosis patients were included. After adjusting the age and gender, twenty non-deformity patients with AS and 20 sagittal deformity patients without AS were selected to compare with patients with AS-related thoracolumbar kyphosis, respectively. Sag angle in deformity patients with AS was smaller than other two groups (*P* < 0.001). No difference was found in Tsv angle and Sacral distance between AS patients with and without deformity. However, these two parameters were shown significant differences between deformity patients with AS and without AS. In deformity patients with AS, no significant differences were found in all parameters between genders Furthermore, there were strong correlations between PT and the bilateral Sag angle (*P* < 0.001).

**Conclusions:**

The thoracolumbar kyphosis secondary to AS affects the Sag angle of the ideal S2AI trajectory which was approximately 20° smaller than that in non-deformity patients with AS, sagittal deformity patients without AS, and the normal population. Additionally, the Tsv angle and the Sacral distance in AS patients with thoracolumbar kyphosis were about 10° and 10 mm larger than those in sagittal deformity patients without AS, and the normal population reported in literatures.

## Introduction

Currently, pelvic fixation is a hot topic in surgery for adult spinal deformity (ASD). Pelvic fixation is required in some specific circumstances such as diabetes, smoking, old age [1], lumbosacral pseudarthrosis [2, 3], severe osteoporosis [4, 5], high body mass index (BMI) [6], revision surgeries for distal junctional kyphosis (DJK)/distal junctional failure (DJF) [7], 3-column osteotomy at the lumbosacral region [8], severe concomitant coronal and sagittal imbalance [1].

Thoracolumbar kyphosis secondary to ankylosing spondylitis (AS) has different spinopelvic characteristics from ASD. In advanced stage of AS, pathological osteogenesis can cause ectopic ossification of soft tissues such as interspinous ligaments and sacroiliac joints [9]. Finally, the pathological change of the lumbosacral spine and sacroiliac joints fuses the spine and pelvis together. Internal fixation to the lower lumbar spine or sacrum can achieve the same surgical outcome of pelvic fixation for most patients with advanced AS [10], while based on our experience, pelvic fixation is mandatory in some specific circumstances: (1) Pseudarthrosis located at L5/S1. Pseudarthrosis is a common complication in AS [11]. If destructed S1 is selected as the lower instrumental vertebra (LIV), loosening and pulling out of screw may occur due to the insufficient strength of internal fixation; (2) Severe lumbar osteoporosis. Osteoporosis is a well-recognized complication of AS [12]. To increase the strength of internal fixation, extending to the pelvis is considered; (3) High BMI. For the same moment arm, distal internal fixations of obese patients suffer greater force than those of the others due to the larger gravity; (4) Revision surgeries. Specially for DJK/DJF; (5) Three-column osteotomy at the lumbosacral region. For example, S1 is selected as LIV after L5 pedicle subtraction osteotomy (PSO). There is only one distal fixation segment that may increase the long-term complication rate; (6) Concomitant coronal and sagittal imbalance. Pelvic fixation may increase strength of internal fixation and avoid the formation of pseudarthrosis; (7) Persisting disease activity. Patients with incomplete sacroiliac joint fusion may have DJF due to persistent inflammatory activity. For these patients, spinopelvic instrumentation through posterior second sacral alar iliac (S2AI) screw fixation is supposed to be an effective and reliable technique [13, 14].

Compared with the traditional sacropelvic fixation technique, previous studies have illustrated that the advantages of less soft tissue dissection and fewer complications following the S2AI screw fixation technique [15–18]. However, the breach rate of S2AI screw placement using free-hand was 8%, even in robotic-guided S2AI screw placement, there still had a breach rate of 4.3% [13, 14]. When a breach happens intraoperatively, neurovascular structures including the superior gluteal artery, the internal iliac vein and artery, the sciatic nerve, the obturator nerve and the lumbosacral plexus are potentially at risk for injury [19]. Hence, to avoid injury, measurement of the mandatory parameters for the ideal S2AI trajectory on CT imaging before surgery is necessary. The data about the ideal S2AI trajectory from previous studies were acquired from a healthy population without spinal deformity [20, 21]. While in most AS patients, sacroiliac ankylosis and rigid thoracolumbar kyphosis may result in the anatomical and functional changes of pelvis which was manifested by the difficulty in identification of the S1 and S2 foramen, and pelvic retroversion driven by kyphotic deformity [22–24]. Therefore, it is of great important to understand the anatomical parameters of the pelvis in AS for the accurate insertion of S2AI screw.

To the best of our knowledge, little is known about whether the thoracolumbar kyphosis secondary to AS affects the S2AI trajectory. There are two primary aims of this study: (1) to evaluate the influence of AS-related thoracolumbar kyphosis on S2AI trajectory parameters; and (2) to compare their S2AI trajectory parameters with those of normal population.

## Materials and methods

### Subjects

This study has been approved by local institutional review board (IRB approved no., 2,011,052). Informed consent was obtained from all patients. AS patients undergoing corrective surgery for thoracolumbar kyphosis from January 2017 to May 2021 were reviewed. All patients were diagnosed with AS according to the Modified New York Criteria [25]. The inclusion criteria were as follows: (1) age older than 18 years; and (2) availability of their pelvic CT images and whole spine standing X-ray lateral views. Patients with previous spinal surgery were excluded from the study. In addition, non-deformity patients with AS and sagittal deformity patients without AS from January 2017 to May 2021, were selected continuously to compare with deformity patients with AS.

### CT reconstruction and radiographic measurements

**Fig. 1:**
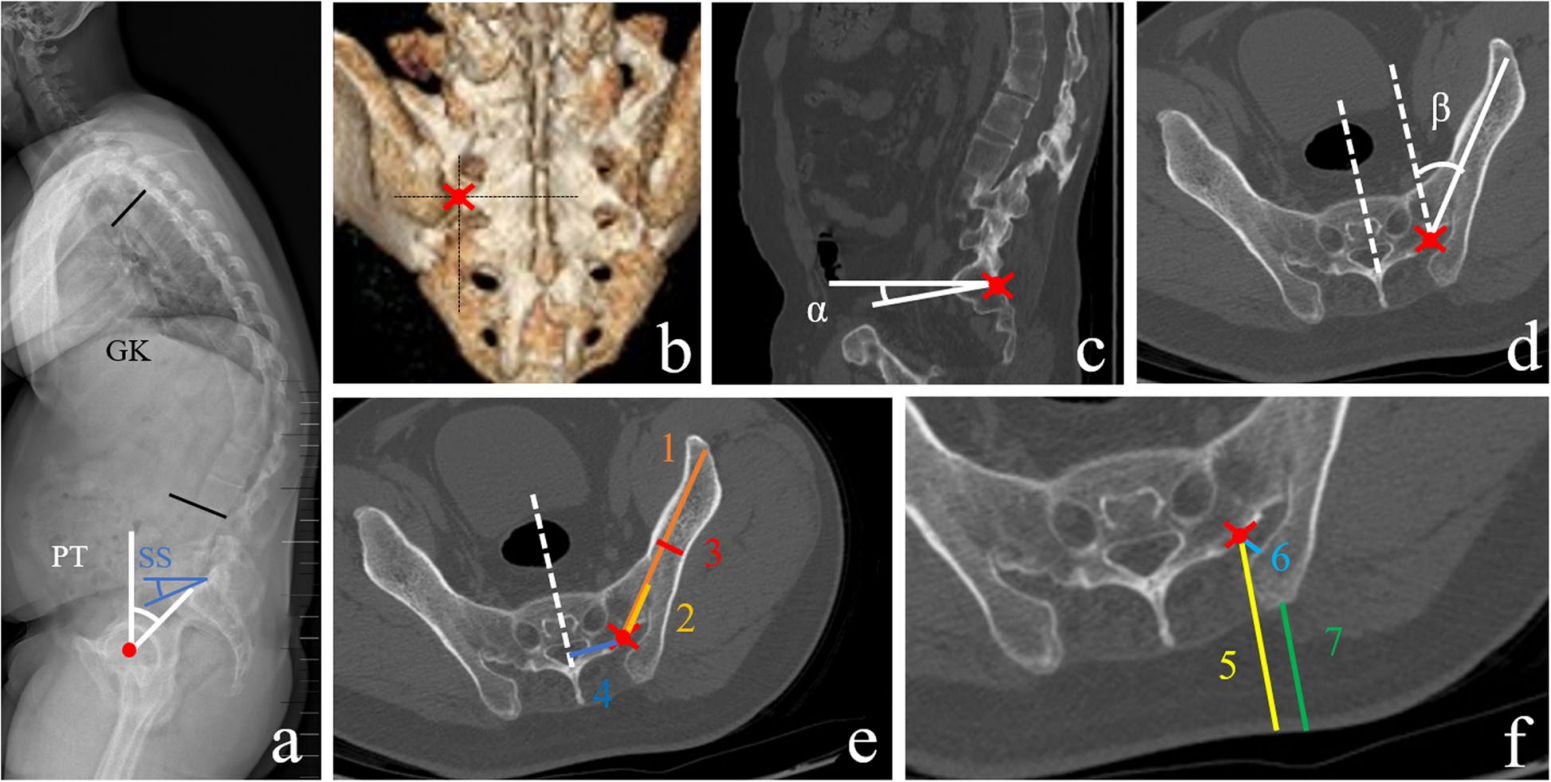
The measurements of sagittal parameters on lateral X-ray and S2AI pathway parameters on CT imaging for AS patients **a** GK, PT and SS were measured on a standard standing lateral radiograph. **b** Three-dimensional reconstruction of the pelvis. The starting point of the S2AI pathway was 1 cm inferior and 1 cm lateral to the S1 dorsal foramen. **c** CT imaging in the sagittal plane. Sag angle (angle α) is determined by the caudal trajectory angulation in the sagittal plane. **d** CT imaging in the transverse plane. Tsv angle (angle β) was defined as lateral trajectory angulation; **e** Max-length (line 1) determined by the maximal distance of the trajectory from S2 ala to the anterior inferior iliac spine; Sacral distance (line 2) was defined as intrasacral trajectory length; Iliac width (line 3) was defined as the narrowest iliac width measured between the inner cortices; S2 midline (line 4) was measured from the starting point lateral to the middle line of S2; **f** Skin distance (line 5) was measured from the starting point lateral to the skin; Iliac wing (line 6) was defined as the distance of the starting point lateral from the nearest iliac wing; PSIS distance (line 7) was measured from PSIS to nearest skin

Measuring methods of parameters were illustrated in Fig. 1. Sagittal radiographic parameters were measured on the X-ray lateral radiographs using Surgimap software (Nemaris, US). Global kyphosis (GK) was measured from the superior endplate of the maximally tilted upper end vertebra to the inferior endplate of the maximally tilted lower end vertebra [26]. Pelvic tilt (PT) was considered as the angle between a vertical line originating at the center of the femoral heads and a line drawn between the same point and middle of the superior endplate of S1. Sacral slope (SS) was the angle between the sacral plate and the horizontal [27] (Fig. 1a).

CT scans of the pelvis from the superior margin of the iliac crest to the lesser trochanter of the femur were obtained. All the patients were in the supine position with the hip in full extension. CT scans were used to investigate the existence of pseudarthrosis, and to evaluate the degree of ossification and pedicle screw trajectory parameters. Brilliance CT 64-channel scanner (Philips Medical Systems, PC Best, Netherlands) was used to scan the whole pelvis with a layer thickness of 5.0 mm, voltage of 120 kV, and current of 320 mA. The reconstruction was performed with a pitch of 1 mm and threshold of 300 HU using Light speed workplace AW4.3 (General electric company, American), a matched CT imaging computer application was used for three-dimensional interactive viewing and manipulation [21]. The S2AI trajectory parameters were measured on CT images. The insertion point of the S2AI screw was 1 cm inferior and 1 cm lateral to the S1 dorsal foramen (Fig. 1b). Ideal S2AI screw trajectories were explored by manipulating CT imaging planes, ensuring that the trajectories were of the greatest length and width of the osseous channel for the patients. Caudal angulation in the sagittal plane (Sag angle), and lateral angulation in the transverse plane (Tsv angle) were measured (Fig. 1c, d). Max-length is defined as the maximal distance of the trajectory from the S2 ala to the anterior inferior iliac spine. Also, the distance of the trajectory in the sacrum was quantified. Iliac width is defined as the narrowest iliac width in the determined trajectory measured between the inner cortices. S2 midline meant the insertion point as measured from the midline of S2 in the coronal view (Fig. 1e). Finally, the distance from the posterior superior iliac spine (PSIS) to the nearest skin, and the distance of the starting point from the nearest skin and iliac wing were measured in the transverse plane, respectively [20] (Fig. 1f). Image parameter measurements were performed by a resident trained in images measurements in 2019. All measurements were repeated 3 times and the average measurement values were used for the final analyses.

### Statistical analysis

Independent t-test was performed with SPSS 19.0 (SPSS Inc., Chicago, IL) to compare the radiographic parameters in deformity with AS group to those in non-deformity with AS group and deformity without AS group, respectively.. It was also used to analyze the morphology of S2AI trajectories between the left and the right side in the pelvis. On account of the differences in morphology between the pelvis of males and females, independent samples t-test was used to detect possible divergence in the above-mentioned data between genders. Data are expressed by mean ± standard deviation. Findings were considered significant when the *P*-value was < 0.05. Correlation analyses were performed using Pearson correlation to demonstrate the relationship between variables.

## Results

**Fig. 2:**
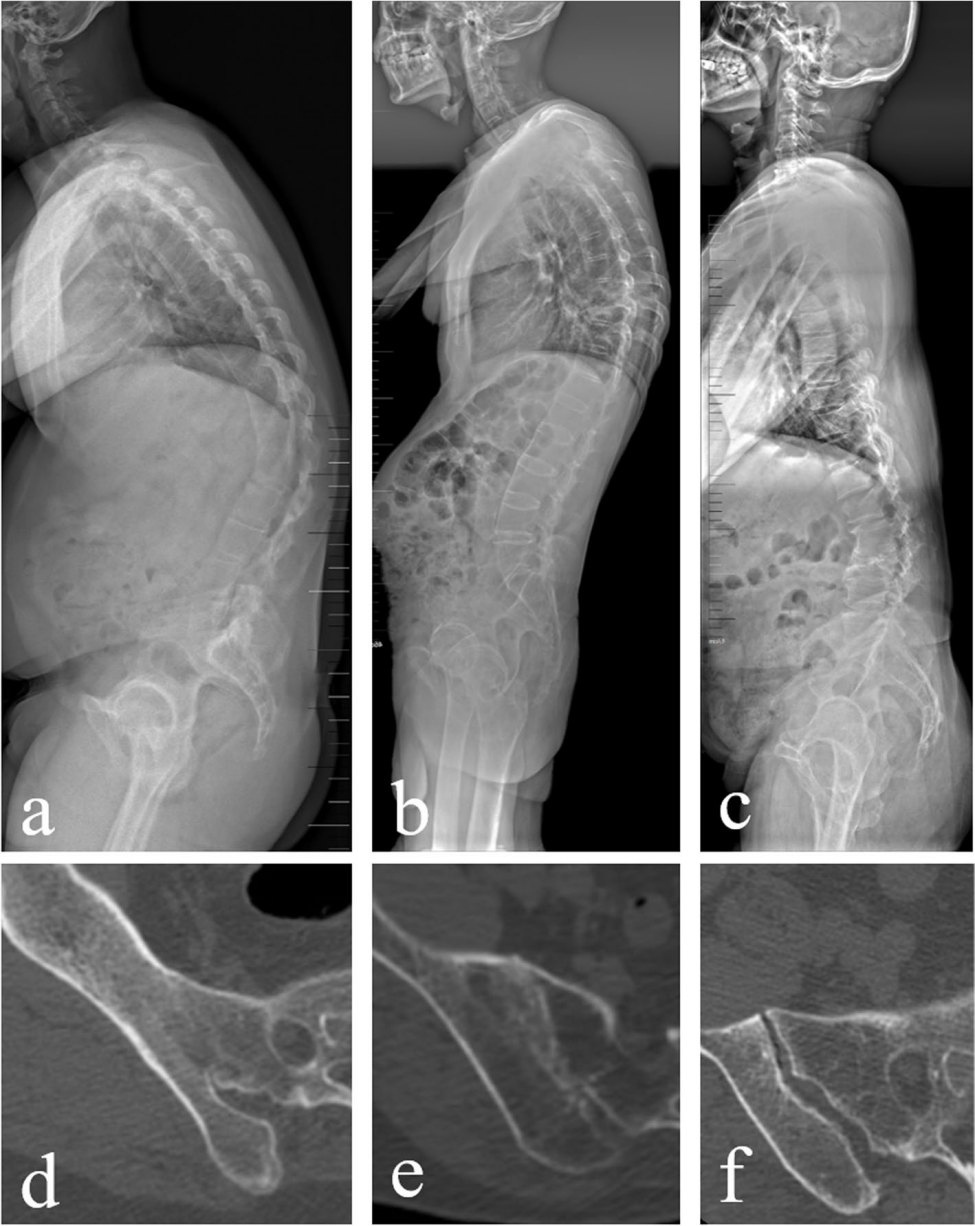
Standing lateral radiograph and CT of sacroiliac joint. **a** AS patient with thoracolumbar kyphosis (GK: 77°). **b** Non-deformity patient with AS. **c** Sagittal deformity patients without AS (posttraumatic kyphotic deformity in thoracolumbar spine). **d-e** CT scans have shown bony fusion in sacroiliac joints of the deformity patient with AS, also the non-deformity patient with AS. **f** No erosion or ankylosis was found in sacroiliac joints of sagittal deformity patients without AS

**Fig. 3:**
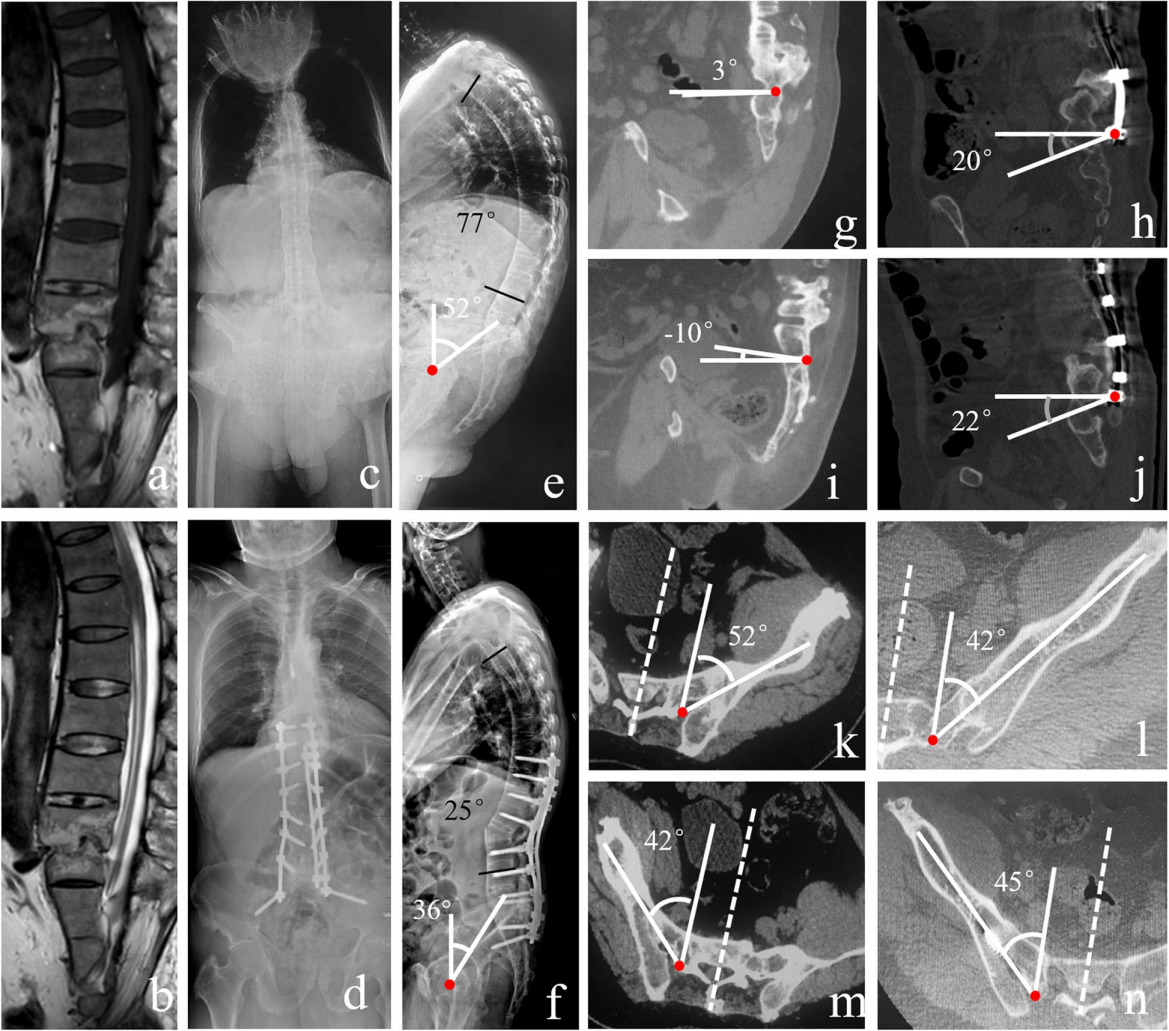
A 55-year-old male AS patient with L5/S1 pseudarthrosis and thoracolumbar kyphosis underwent L2 PSO combined with bilateral S2AI screw fixation **a-b** A L5/S1 pseudarthrosis was shown on the MR images (a: T1-weighted; b: T2-weighted). **c-d** Coronal view. **e-f** On the standing lateral radiograph, the GK (77° vs 25°) and PT (52° vs 36°) of the patient were improved after surgery. **g-j** The lateral view of S2AI trajectory on the CT imaging before and after surgery on both sides. **k-n** The transverse view of S2AI trajectory on CT imaging

Table 1 showed the details of the patients. Ninety-four AS patients with thoracolumbar kyphosis were included in the current study. For comparison, 20 non-deformity patients with AS and 20 sagittal deformity patients without AS were selected after adjusting the age and gender (Fig. 2). One of the deformity patients with AS underwent a 1-level pedicle subtraction osteotomy (PSO) combined with bilateral S2AI screw fixation (Fig. 3). Significant difference was shown in PT (37.7 ± 10.9 vs 28.1 ± 15.4, *P* < 0.001) and SS (6.7 ± 8.4 vs 19.0 ± 11.3, *P* = 0.001) between AS deformity group and non-AS deformity group. No significant difference was found in body mass index (BMI) among the three groups.

**Table 1:** Details of the patients

	AS deformity	AS non-deformity	non-AS deformity
n	94	20	20
Gender, male (%)	89	90	90
age	36.8 ± 9.8	37.5 ± 10.9	40.8 ± 5.1
BMI	25.9 ± 5.3	24.5 ± 3.5	24.6 ± 3.4
GK	69.5 ± 15.9	46.2 ± 17.7*	65.7 ± 14.9
PT	37.7 ± 10.9	24.5 ± 11.2*	28.1 ± 15.4*
SS	6.7 ± 8.4	18.4 ± 8.9*	19.0 ± 11.3*

*AS* Ankylosing spondylitis, *BMI* Body mass index, *GK* Global kyphosis, *PT* Pelvic tilt, *SS* Sacral slope

* data was statistically different from that of AS deformity (*P* < 0.05)

The ideal S2AI screw trajectories could be found in each pelvis through 3D radiographic analysis. The left and right screw trajectories parameters of three types of patients were summarized in Table 2. The average Sag angle was found to be smaller in deformity patients with AS than those in other two groups (*P* < 0.001). Although there was no significant difference in Tsv angle between deformity and non-deformity patients with AS, Tsv angle was larger in deformity patients with AS than that in sagittal deformity patients without AS (*P* < 0.001). Similar result could be seen in the value of the Sacral distance (*P* < 0.001).

**Table 2:** Bilateral S2AI screw trajectory parameters on CT imaging

Parameters	Left			Right		
	AS deformity	AS non-deformity	non-AS deformity	AS deformity	AS non-deformity	non-AS deformity
Sag angle (°)	8.2 ± 11.7	26.6 ± 6.2*	26.9 ± 10.2*	8.0 ± 13.3	27.6 ± 6.9*	27.0 ± 10.5*
Tsv angle (°)	42.6 ± 6.7	43.1 ± 6.1	36.8 ± 7.3*	43.1 ± 7.7	42.5 ± 6.3	36.9 ± 7.5*
Max-length (mm)	111.6 ± 13.8	111.8 ± 11.3	114.4 ± 7.5	112.3 ± 14.7	112.9 ± 9.7	114.1 ± 7.8
Sacral distance (mm)	36.4 ± 6.1	34.9 ± 7.6	30.6 ± 6.6*	38.7 ± 5.8	38.2 ± 4.8	30.6 ± 5.8*
Iliac width (mm)	14.4 ± 4.0	12.3 ± 4.0	13.4 ± 2.0	14.3 ± 4.2	12.0 ± 1.9	13.6 ± 1.7
S2 midline (mm)	26.3 ± 3.6	25.4 ± 4.1	25.8 ± 6.4	26.4 ± 4.1	25.4 ± 2.6	25.7 ± 6.3
Skin distance (mm)	41.4 ± 14.8	36.8 ± 8.1	37.6 ± 9.9	41.0 ± 14.1	37.8 ± 5.7	37.2 ± 9.4
Iliac wing (mm)	12.7 ± 3.8	13.4 ± 4.1	12.5 ± 2.4	13.0 ± 4.5	13.2 ± 2.6	12.9 ± 2.0
PSIS distance (mm)	20.6 ± 12.3	20.1 ± 6.4	21.9 ± 9.1	20.1 ± 12.6	21.0 ± 6.3	21.3 ± 8.5

*PSIS* Posterior superior iliac spine

* data was statistically different from that of AS deformity (*P* < 0.05)

The results of the correlational analysis were presented in Table 3. There were strong correlations between PT and the bilateral Sag angle (*P* < 0.001). Meanwhile, there were weak correlations between GK and the bilateral Sag angle (L: *P* = 0.030; R: *P* = 0.035). Additionally, no significant correlation was found between the Sacral distance and GK, as well as PT. A weak correlation was found between SS and Sag angle (L: *P* = 0.026; R: *P* = 0.006).

**Table 3:** Correlation between sagittal parameters on lateral X-ray radiographs and the ideal S2AI screw trajectory parameters on CT imaging in deformity patients with AS

*Parameters*		*Left*			*Right*		
		GK	PT	SS	GK	PT	SS
*Sag angle*	*r*	−0.227^a^	− 0.618^b^	0.232^a^	− 0.220^a^	− 0.607^b^	0.284^b^
*Tsv angle*	*r*	0.080	−0.243^a^	0.052	0.004	−0.239^a^	0.131
*Max-length*	*r*	−0.192	− 0.137	− 0.002	−0.304^b^	− 0.176	−0.14
*Sacral distance*	*r*	−0.023	0.133	−0.084	0.026	0.103	−0.089
*Iliac width*	*r*	0.043	0.458^b^	−0.103	0.062	0.324^b^	−0.096
*S2 midline*	*r*	0.169	0.039	−0.079	0.133	0.445^b^	−0.098
*Skin distance*	*r*	0.148	−0.038	−0.238^a^	0.113	−0.053	− 0.250^a^
*Iliac wing*	*r*	0.018	0.162	−0.139	−0.007	0.178	−0.052
*PSIS distance*	*r*	0.062	−0.135	−0.201	0.063	−0.109	− 0.166

*PT* Pelvic tilt, *GK* Global kyphosis, *PSIS* Posterior superior iliac spine

^a^Significant correlation was established at the 0.05 level

^b^Significant correlation was established at the 0.01 level

The data from different genders were also summarized, respectively (Table 4). No statistical difference between genders in the mean Sag angle was found. In the meantime, there was no conspicuous difference existed in the average Tsv angle between genders. Also, no significant difference was found in the Sacral distance between genders.

**Table 4:** Comparison of the bilateral S2AI screw trajectory parameters between genders in deformity patients with AS

Parameters	Left			Right		
	*Male*	*Female*	*P*	*Male*	*Female*	*P*
Sag angle (°)	8.0 ± 11.2	9.6 ± 15.5	0.096	7.8 ± 12.9	9.4 ± 16.6	0.254
Tsv angle (°)	42.3 ± 6.6	44.3 ± 7.9	0.389	42.7 ± 7.5	46.0 ± 9.6	0.269
Max-length (mm)	111.7 ± 13.6	111.0 ± 16.0	0.417	112.7 ± 13.6	108.8 ± 20.6	0.389
Sacral distance (mm)	36.2 ± 5.8	37.8 ± 8.9	0.081	38.9 ± 5.5	36.7 ± 8.0	0.262
Iliac width (mm)	14.6 ± 4.0	12.5 ± 4.1	0.867	14.6 ± 4.2	12.3 ± 4.4	0.711
S2 midline (mm)	26.2 ± 3.6	27.3 ± 3.3	0.359	26.2 ± 3.9	27.2 ± 5.7	0.119
Skin distance (mm)	41.6 ± 14.6	40.4 ± 17.0	0.381	41.1 ± 14.0	39.7 ± 15.2	0.915
Iliac wing (mm)	12.8 ± 4.0	11.6 ± 2.0	0.209	12.8 ± 4.5	15.0 ± 3.8	0.991
PSIS distance (mm)	20.3 ± 12.0	22.9 ± 14.9	0.151	19.8 ± 12.4	22.1 ± 14.9	0.162

*PSIS* Posterior superior iliac spine

## Discussion

Although S2AI screw technique is an increasingly popular method of spinopelvic fixation in spinal deformity surgery, the potential risk of neurovascular injury should not be ignored. The evaluation of preoperative S2AI trajectory parameters is a major step to reduce the incidence of complications. Shillingford et al. reported that the ideal S2AI trajectory was aimed at caudally 20° to 25° in the sagittal plane [14]. However, this S2AI trajectory parameter was based on the normal population [20, 21, 28, 29], and the application of S2AI screw fixation in AS patients has not been reported before. Multiple studies illustrated that AS patients and the normal population were found to be significantly different in terms of sagittal spinopelvic parameters on radiography, especially PT [21, 23, 30, 31]. In these studies, the average PT in AS patients was much larger than that in the normal population. When performing the insertion of S2AI screws for AS patients, pelvic backward rotation can not be ignored because of the potential variations of S2AI trajectory in AS patients. If AS patient was performed using S2AI fixation according to the ideal trajectory for the normal population, it is likely to cause a larger Sag angle of screw insertion leading to a breach. Subsequently, sciatic nerve and superior/inferior gluteal artery and vein injury may occur due to the breach. Moreover, navigation and robotics have become increasingly popular, which may facilitate the insertion of screws. However, these medical equipments are not available in hospitals in less developed countries and regions. To date, little is known about the influence of AS-related thoracolumbar kyphosis on the S2AI trajectory parameters. Therefore, the current study aimed to establish the morphometric characteristics of S2AI screws fixation in AS patients with thoracolumbar kyphosis.

In the present study, a valuable finding that no significant difference in the ideal S2AI trajectory parameters between genders was found, was consistent with the results reported by Shillingford et al. [14]. Moreover, three S2AI trajectory parameters, including the Sag angle, Tsv angle, Sacral distance, of deformity patients with AS were found to be different compared with those of sagittal deformity patients without AS. Similar results were found when compared them with those of the normal population reported in literatures. Data from previous studies revealed that the Sag angle in males and females was respectively 26.0–36.7° and 23.0–41.6° on the left side, 27.9–37.4° and 32.4–41.9° on the right side [20, 21, 28, 29]. However, the average Sag angle was much smaller in the AS patients with thoracolumbar kyphosis than that in the normal population (Male: L:8.0°; R:7.8° vs Female: L:9.6°; R:9.4°). Another valuable result was shown that the Sag angle of the ideal S2AI trajectory had a strong correlation to PT of the patient (L: *r* = − 0.618, *P* < 0.001; R: *r* = − 0.607, *P* < 0.001). These results indicated that most AS patients with thoracolumbar kyphosis were characterized by pelvic retroversion (Fig. 2a). A previous study had illustrated that the average PT in deformity patients with AS was 38.5°, much larger than 11.1° in young adult volunteers [22]. Another study performed by Shin et al. showed that AS patients exhibited more PT than the normal controls (20.0° to 13.5°) [23]. Moreover, although a correlation between SS and the Sag angle was also found, it was weaker than that between PT and the Sag angle. Therefore, in deformity patients with AS, the Sag angle of the ideal S2AI trajectory is associated with the PT.

Interestingly, another valuable finding that the mean Sacral distance and Tsv angle were larger in deformity patients with AS than those in sagittal deformity patients without AS. This finding was also inconsistent with the results of the prior study which reported that it would be at 26.2-28 mm before the ilium is reached [21]. Also, compared with that of the normal population, the mean Tsv angle of AS patients was larger (AS: 42.7–46.0° vs Normal: 35.7–37.2°). Unlike the Sag angle, the results of correlation analysis indicated that the Tsv angle and Sacral distance were not primarily affected by the thoracolumbar kyphosis secondary to AS. These inconsistencies might be explained by the fact that the direction of the ilium on both sides and the distance before piercing the sacroiliac joint were affected by the biologic fusion of the sacroiliac joint in AS patients (Fig. 2d-e). Yao et al. reported that part of the dorsal sacrum was covered by the ilium due to the biologic fusion of the sacroiliac joint [24]. This could also be demonstrated by the results that no difference was shown in the Sacral distance and Tsv angle in AS patients with and without deformity. Except for the Sag angle, Tsv angle and Sacral distance, other S2AI trajectory parameters of AS patients were close to those of the normal population in other studies. Consequently, the effect of AS-related thoracolumbar kyphosis on the ideal S2AI trajectory is primarily functional rather than anatomical.

Furthermore, based on a previous study, screws typically range from 7.5 to 9.5 mm in diameter and range 50 to 75 mm in length [14, 32], which can apply to the S2AI screw fixation in AS patients with thoracolumbar kyphosis. One of the deformity patients with AS in this study underwent PSO combined with bilateral S2AI screw fixation and obtained satisfactory surgical outcomes (Fig. 3). However, compared with that in normal population [14], the trajectory for the S2AI screw in AS patient was aimed more laterally (approximately 5–10°) in the transverse plane and more cranially (about 20°) in the sagittal plane.

Nevertheless, the results of this study need to be taken in light of certain limitations. Firstly, a bias may exist due to the radiographic parameters was not measured by another investigator. Secondly, because CT scans were used preoperatively, the radiation exposure of patients should not be ignored. However, CT scans may be required because they are helpful for the evaluation of pseudarthrosis. Future prospective, results of the multicenter studies with larger sample sizes are required to definite the correlation of the AS-related thoracolumbar kyphosis and the ideal S2AI trajectory parameters. Thirdly, it is well-known that patients with deformity frequently shows high PT compared with the normal populations. However, PT and SS was different between deformity patients with and without AS in current study. Therefore, the influence of non-AS kyphosis on parameters can not be completely ignored.

## Conclusions

There were no significant differences in the S2AI trajectory parameters of AS patients with thoracolumbar kyphosis between genders. Compared with that in sagittal deformity patients without AS, non-deformity patients with AS and the normal population, the preoperative Sag angle of the S2AI trajectory was approximately 20° smaller in AS patients with thoracolumbar kyphosis because of the pelvic retroversion. Besides, the Tsv angle and the Sacral distance of the S2AI trajectory in AS patients with thoracolumbar kyphosis were about 10° and 10 mm larger than those in the normal population probably due to biologic fusion of the sacroiliac joint. To sum up, the thoracolumbar kyphosis secondary to AS affects the morphometric characteristics of S2AI screws fixation functionally. In the absence of navigation and robotics, the S2AI screw fixation is still an alternative and feasible method for thoracolumbar kyphosis secondary to AS patients who require sacroiliac fixation.

## Data Availability

All data generated or analyzed during this study are included in this article. We confirm that the Availability of data and materials refers to the raw data generated and used for this study. Meanwhile, the datasets used and/or analyzed during the current study are available from the corresponding author on reasonable request.

## Abbreviations

AS: Ankylosing spondylitis
ASD: Adult spinal deformity
BMI: High body mass index
DJF: Distal junctional failure
DJK: Distal junctional kyphosis
GK: Global kyphosis
LIV: Lower instrumental vertebra
LL: Lumbar lordosis
PSO: Pedicle subtraction osteotomy
PSIS: Posterior superior iliac spine
PT: Pelvic tilt
S2AI: Second sacral alar iliac
SS: Sacral slope

## References

[1] Shen FH, Mason JR, Shimer AL, Arlet VM (2013). Pelvic fixation for adult scoliosis. Eur Spine J.

[2] Mattei TA, Fassett DR (2013). Combined S-1 and S-2 sacral alar-iliac screws as a salvage technique for pelvic fixation after pseudarthrosis and lumbosacropelvic instability: technical note. J Neurosurg Spine.

[3] Smith EJ, Kyhos J, Dolitsky R, Yu W, O'Brien J (2018). S2 alar iliac fixation in long segment constructs, a two- to five-year follow-up. Spine Deformity..

[4] Gotis-Graham I, McGuigan L, Diamond T, Portek I, Quinn R, Sturgess A, Tulloch R (1994). Sacral insufficiency fractures in the elderly. J Bone Joint Surg Br.

[5] Dasgupta B, Shah N, Brown H, Gordon TE, Tanqueray AB, Mellor JA (1998). Sacral insufficiency fractures: an unsuspected cause of low back pain. Br J Rheumatol.

[6] Salzmann SN, Ortiz Miller C, Carrino JA, Yang J, Shue J, Sama AA, Cammisa FP, Girardi FP, Hughes AP (2019). BMI and gender increase risk of sacral fractures after multilevel instrumented spinal fusion compared with bone mineral density and pelvic parameters. Spine J.

[7] Bridwell KH (2005). Utilization of iliac screws and structural interbody grafting for revision spondylolisthesis surgery. Spine (Phila Pa 1976).

[8] Suk SI, Chung ER, Lee SM, Lee JH, Kim SS, Kim JH (2005). Posterior vertebral column resection in fixed lumbosacral deformity. Spine (Phila Pa 1976).

[9] Chang KW, Tu MY, Huang HH, Chen HC, Chen YY, Lin CC (2006). Posterior correction and fixation without anterior fusion for pseudoarthrosis with kyphotic deformity in ankylosing spondylitis. Spine (Phila Pa 1976).

[10] Huang JC, Diao WY, Qian BP, Wang B, Yu Y, Qiao M, Qiu Y (2020). Can fusion to S1 maintain favorable surgical outcomes following one-level pedicle subtraction osteotomy in patients with thoracolumbar kyphosis secondary to ankylosing spondylitis?. Eur Spine J.

[11] Kim SK, Shin K, Song Y, Lee S, Kim TH (2016). Andersson lesions of whole spine magnetic resonance imaging compared with plain radiography in ankylosing spondylitis. Rheumatol Int.

[12] Klingberg E, Nurkkala M, Carlsten H, Forsblad-d’Elia H (2014). Biomarkers of bone metabolism in ankylosing spondylitis in relation to osteoproliferation and osteoporosis. J Rheumatol.

[13] Laratta JL, Shillingford JN, Lombardi JM, Alrabaa RG, Benkli B, Fischer C, Lenke LG, Lehman RA (2018). Accuracy of S2 alar-iliac screw placement under robotic guidance. Spine Deformity.

[14] Shillingford JN, Laratta JL, Tan LA, Sarpong NO, Lin JD, Fischer CR, Lehman RA, Kim YJ, Lenke LG (2018). The free-hand technique for S2-alar-iliac screw placement: a safe and effective method for sacropelvic fixation in adult spinal deformity. J Bone Joint Surg Am.

[15] Mazur MD, Mahan MA, Shah LM, Dailey AT (2017). Fate of S2-alar-iliac screws after 12-month minimum radiographic follow-up: preliminary results. Neurosurgery.

[16] O'Brien JR, Yu W, Kaufman BE, Bucklen B, Salloum K, Khalil S, Gudipally M (2013). Biomechanical evaluation of S2 alar-iliac screws: effect of length and quad-cortical purchase as compared with iliac fixation. Spine (Phila Pa 1976).

[17] Sponseller PD, Zimmerman RM, Ko PS, Pull Ter Gunne AF, Mohamed AS, Chang TL, Kebaish KM (2010). Low profile pelvic fixation with the sacral alar iliac technique in the pediatric population improves results at two-year minimum follow-up. Spine (Phila Pa 1976).

[18] Elder BD, Ishida W, Lo SL, Holmes C, Goodwin CR, Kosztowski TA, Bydon A, Gokaslan ZL, Wolinsky JP, Sciubba DM (2017). Use of S2-alar-iliac screws associated with less complications than iliac screws in adult lumbosacropelvic fixation. Spine (Phila Pa 1976).

[19] Abdul-Jabbar A, Yilmaz E, Iwanaga J, Tawfik T, O'Lynnger TM, Schildhauer TA, Chapman J, Oskouian RJ, Tubbs RS (2018). Neurovascular relationships of S2AI screw placement: anatomic study. World Neurosurg.

[20] Chang TL, Sponseller PD, Kebaish KM, Fishman EK (2009). Low profile pelvic fixation: anatomic parameters for sacral alar-iliac fixation versus traditional iliac fixation. Spine (Phila Pa 1976).

[21] Zhu F, Bao HD, Yuan S, Wang B, Qiao J, Zhu ZZ, Liu Z, Ding YT, Qiu Y (2013). Posterior second sacral alar iliac screw insertion: anatomic study in a Chinese population. Eur Spine J.

[22] Debarge R, Demey G, Roussouly P (2011). Sagittal balance analysis after pedicle subtraction osteotomy in ankylosing spondylitis. Eur Spine J.

[23] Shin JK, Lee JS, Goh TS, Son SM (2014). Correlation between clinical outcome and spinopelvic parameters in ankylosing spondylitis. Eur Spine J.

[24] Yao Z, Zheng G, Zhang Y, Wang Z, Zhang X, Cui G, Wang Y (2016). Selection of lowest instrumented vertebra for thoracolumbar kyphosis in ankylosing spondylitis. Spine (Phila Pa 1976).

[25] van der Linden S, Valkenburg HA, Cats A (1984). Evaluation of diagnostic criteria for ankylosing spondylitis. A proposal for modification of the New York criteria. Arthritis Rheum.

[26] Qian BP, Wang XH, Qiu Y, Wang B, Zhu ZZ, Jiang J, Sun X (2012). The influence of closing-opening wedge osteotomy on sagittal balance in thoracolumbar kyphosis secondary to ankylosing spondylitis: a comparison with closing wedge osteotomy. Spine (Phila Pa 1976).

[27] Boulay C, Tardieu C, Hecquet J, Benaim C, Mouilleseaux B, Marty C, Prat-Pradal D, Legaye J, Duval-Beaupere G, Pelissier J (2006). Sagittal alignment of spine and pelvis regulated by pelvic incidence: standard values and prediction of lordosis. Eur Spine J.

[28] O'Brien JR, Matteini L, Yu WD, Kebaish KM (2010). Feasibility of minimally invasive sacropelvic fixation: percutaneous S2 alar iliac fixation. Spine (Phila Pa 1976).

[29] O'Brien JR, Yu WD, Bhatnagar R, Sponseller P, Kebaish KM (2009). An anatomic study of the S2 iliac technique for lumbopelvic screw placement. Spine (Phila Pa 1976).

[30] Debarge R, Demey G, Roussouly P (2010). Radiological analysis of ankylosing spondylitis patients with severe kyphosis before and after pedicle subtraction osteotomy. Eur Spine J.

[31] Lee JS, Suh KT, Kim JI, Goh TS (2014). Analysis of sagittal balance of ankylosing spondylitis using spinopelvic parameters. J Spinal Disord Tech.

[32] Peelle MW, Lenke LG, Bridwell KH, Sides B (2006). Comparison of pelvic fixation techniques in neuromuscular spinal deformity correction: Galveston rod versus iliac and lumbosacral screws. Spine (Phila Pa 1976).

